# Whole‐genome resequencing‐based QTL‐seq identified candidate genes and molecular markers for fresh seed dormancy in groundnut

**DOI:** 10.1111/pbi.13266

**Published:** 2019-10-20

**Authors:** Rakesh Kumar, Pasupuleti Janila, Manish K. Vishwakarma, Aamir W. Khan, Surendra S. Manohar, Sunil S. Gangurde, Murali T. Variath, Yaduru Shasidhar, Manish K. Pandey, Rajeev K. Varshney

**Affiliations:** ^1^ International Crops Research Institute for the Semi‐Arid Tropics (ICRISAT) Hyderabad India; ^2^ Borlaug Institute for South Asia (BISA) CIMMYT Jabalpur India

**Keywords:** abscisic acid, candidate genes, groundnut, fresh seed dormancy, QTL‐seq, RILs, single nucleotide polymorphism, whole‐genome sequencing

## Abstract

The subspecies *fastigiata* of cultivated groundnut lost fresh seed dormancy (FSD) during domestication and human‐made selection. Groundnut varieties lacking FSD experience precocious seed germination during harvest imposing severe losses. Development of easy‐to‐use genetic markers enables early‐generation selection in different molecular breeding approaches. In this context, one recombinant inbred lines (RIL) population (ICGV 00350 × ICGV 97045) segregating for FSD was used for deploying QTL‐seq approach for identification of key genomic regions and candidate genes. Whole‐genome sequencing (WGS) data (87.93 Gbp) were generated and analysed for the dormant parent (ICGV 97045) and two DNA pools (dormant and nondormant). After analysis of resequenced data from the pooled samples with dormant parent (reference genome), we calculated delta‐SNP index and identified a total of 10,759 genomewide high‐confidence SNPs. Two candidate genomic regions spanning 2.4 Mb and 0.74 Mb on the B05 and A09 pseudomolecules, respectively, were identified controlling FSD. Two candidate genes—*RING‐H2 finger protein* and *zeaxanthin epoxidase—*were identified in these two regions, which significantly express during seed development and control abscisic acid (ABA) accumulation. QTL‐seq study presented here laid out development of a marker, GMFSD1, which was validated on a diverse panel and could be used in molecular breeding to improve dormancy in groundnut.

## Introduction

The life begins in most of the higher plants through seed and regulation of its germination plays a crucial role for plant survival, particularly during unfavourable environmental conditions (Shu *et al*., 2015). Seed dormancy and germination are highly coordinated molecular processes which influence the crop productivity in both cereals and legumes in two ways (a) uneven seed germination at the time point of sowing and (b) *in situ* seed germination during harvesting, notably both affect the seed quality and crop yield (Finch‐Savage and Leubner‐Metzger, 2006; Penfield, 2017). Therefore, fresh seed dormancy (FSD) is one of the most important traits that control the initial short period of dormancy in the freshly matured or harvested seed/kernel.

Groundnut (*Arachis hypogaea*) is an important grain legume and oilseed crop a key role in the human nutrition. Groundnut is an allotetraploid crop with a genome size of 2.7 Gbp and was domesticated in South and Central America from its wild ancestral species *A. duranensis* and *A. ipaensis* (Bertioli *et al*., 2016; Chen *et al*., 2016). The sequencing of both the subspecies of cultivated tetraploid groundnut along with other diverse accessions provided greater insights of evolution and domestication (Bertioli *et al*., 2019; Chen *et al*., 2019; Zhuang *et al*., 2019). In Asia and Africa, groundnut is grown as major legume crop. During 2017, the annual world groundnut production was more than 47 million tons of which Asia contributed 62.5% (FAOSTAT, 2017). Particularly, most of the varieties grown in the Asia are Spanish type that lack fresh seed dormancy have significant economic losses due to *in-situ* germination of seeds during harvest, which affect economic strength of small holder farmers. In addition, this reduces the quality of groundnut seeds, thereby limiting their end use and global trade. As an option, foliar spray of maleic hydrazide (growth retardant) has been used to accelerate dormancy in groundnut (Gupta *et al*., 1985), which is not an economical substitute. Therefore, it is important to cultivate varieties that significantly possess 2–3 weeks of FSD for sustainable agricultural benefit for smallholder farmers and industries.

In recent years, significant efforts have been made in cereals to understand seed dormancy trait in order to reduce the yield loss and the kernel quality caused due to preharvest sprouting (PHS) or *in situ* seed germination (Gao and Ayele, 2014; Nakamura, 2018). As a result, several quantitative trait loci (QTLs) and candidate genes for PHS have been identified in wheat (Li *et al*., 2004; Nakamura, 2018; Ogbonnaya *et al*., 2008), rice (Lee *et al*., 2017; Li *et al*., 2004) and barley (Li *et al*., 2003, 2004; Nakamura, 2018). Earlier, a gene *GA20-oxidase* was also identified as a candidate gene in the QTL region controlling PHS in rice (Li *et al*., 2004). Nevertheless, after more than a decade of hard work, causal genes associated with the seed dormancy and PHS have been identified in wheat and barley. These genes include *alanine aminotransferase* (*AlaAT*) and *mitogen‐activated Protein Kinase Kinase 3* (*MKK3*) in barley (Nakamura *et al*., 2016; Sato *et al*., 2016) and *Phs1* and *mother of FT* and *TFL1* (*MFT*) in wheat (Nakamura *et al*., 2011; Torada *et al*., 2016). Although it has been reported that groundnut germplasm possesses significant diversity for seed dormancy (Issa *et al*., 2010; Nautiyal *et al*., 2001; Wang *et al*., 2012; Yaw *et al*., 2008), it is worth mentioning that such detailed studies on FSD in groundnut are lacking (Silva *et al*., 2017; Vishwakarma *et al*., 2016).

Genomics‐assisted breeding (GAB) can significantly shorten the breeding cycle time for the improvement of elite cultivars with desired traits (Varshney *et al*., 2015, 2018a,b). Notably, for the fruitful GAB, primary requirement is identification of marker tightly linked with the desired trait(s). Although Vishwakarma *et al*. (2016) reported two major QTLs controlling FSD, however, the use of F_2_ generation with limited multiseason phenotyping does not help in precise detection of candidate genomic regions. With the availability of draft genome sequences of the diploid progenitors and tetraploid cultivated groundnut (Bertioli *et al*., 2016; Chen *et al*., 2016; Bertioli *et al*., 2019; Chen *et al*., 2019; Zhuang *et al*., 2019), candidate gene discovery and marker development have become more precise and reliable. Of the available sequencing‐based approaches, QTL‐seq approach offers great benefits by identifying genomic region(s) and candidate genes leading to development of diagnostic markers. This approach has already been deployed successfully in some legume crops including groundnut for foliar disease (rust and LLS) resistance (Pandey *et al*., 2017), shelling percentage (Luo *et al*., 2019a), bacterial wilt (Luo *et al*., 2019b) and test a colour (Zhao *et al*., 2019). QTL‐seq approach has also been successfully deployed in discovery of genomic regions and candidate genes with high accuracy and precision in some other crops such as cucumber (Lu *et al*., 2014), tomato (Illa‐Berenguer *et al*., 2015), pigeonpea (Singh *et al*., 2016a) and chickpea (Das *et al*., 2015; Singh *et al*., 2016b). In view of above, we have used RIL population (ICGV 00350 × ICGV 97045) for performing whole‐genome sequencing of pooled samples from contrasting phenotypes and dormant parent followed by QTL‐seq analysis. This study has identified candidate genomic regions and genes, and reports development of molecular markers for FSD in groundnut.

## Results

### Phenotyping and construction of pools

**Figure 1:**
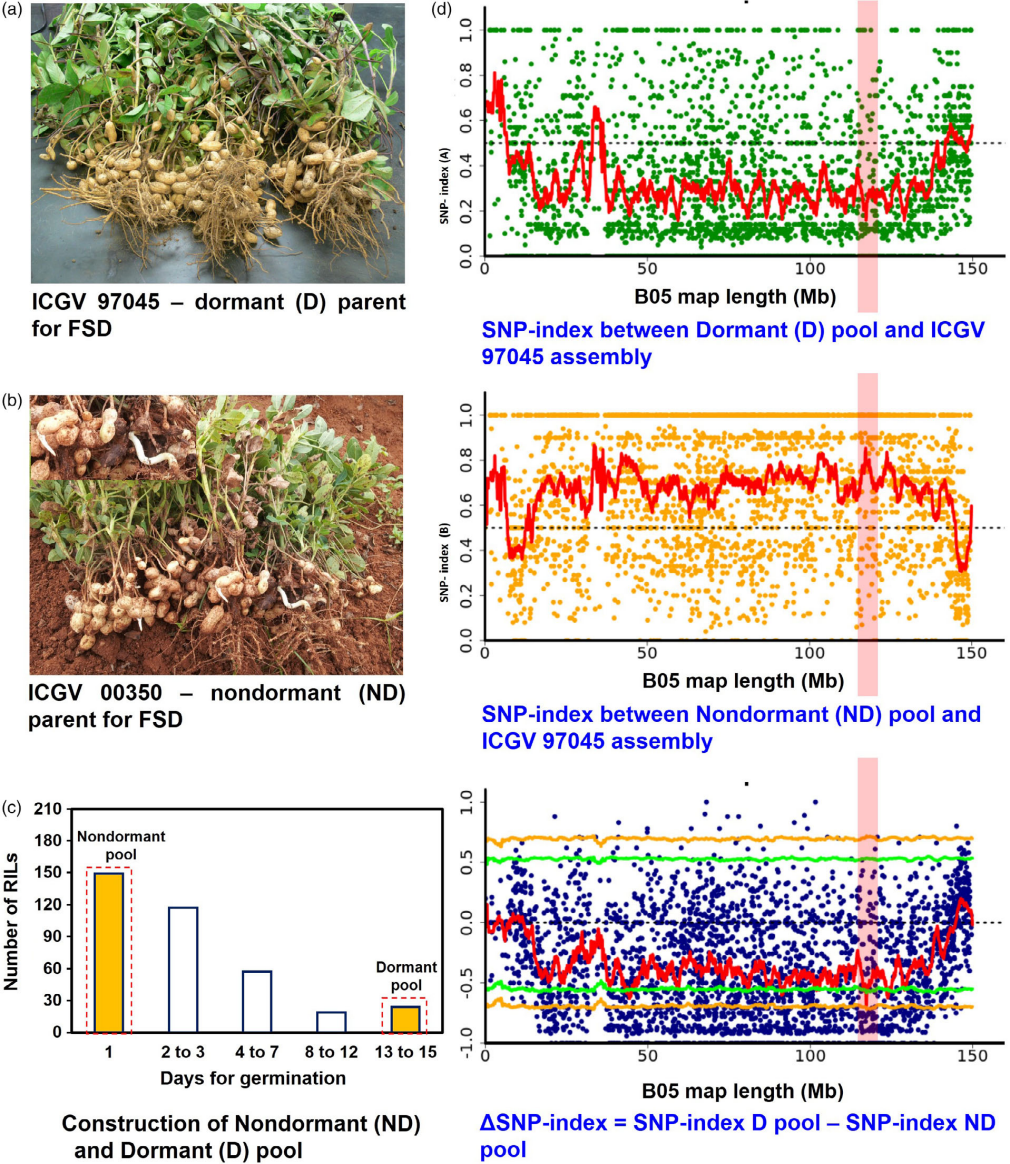
A QTL‐seq approach to identify genomic regions controlling fresh seed dormancy (FSD). (a) ICGV 97045: dormant parent for FSD; (b) ICGV 00350: nondormant parent for FSD; (c) Phenotypic segregation for FSD phenotype in F_2_, dormant lines were progressed for RIL development. The DNA of 20 RILs with extreme phenotypes (dormant and nondormant) was used to develop dormant (D) and nondormant (ND) pools; (d) SNP index plot between D pool and ICGV 97045 assembly (top), ND pool and ICGV 97045 assembly (middle) and ∆SNP index plot (bottom) of pseudomolecule B05 with statistical confidence interval under the null hypothesis of no QTLs (orange, *P* < 0.01; and green, *P* < 0.05). The significant genomic region identified for FSD is shaded (114.45–116.69 Mb).

The RIL population (ICGV 00350 × ICGV 97045) used in this study had high phenotypic variability for FSD. The dormant parent ICGV 97045 was used as source for dormancy, which has FSD up to 15 days, whereas ICGV 00350 was used as nondormant parent which germinate within 48 hours. The previous genetic mapping effort using a subset (368) of large F_2‐3_ population (>800) performed destructive method of phenotyping and showed clear trait segregation (Vishwakarma *et al*., 2016). Therefore, we followed then SSD for rest of the F_2‐3_ lines and finally developed RIL population with 366 lines. Among 366 RILs, 149 lines showed nondormant phenotype (seeds germinated within 24 h), 117 lines showed FSD between 2 and 3 days, 57 lines showed FSD up to 4–7, and 19 showed FSD up to 8–12 days. Interestingly, only 24 lines showed dormancy up to 13–15 days (Figure 1c and Table S1, S2). Later, the RILs with extreme phenotypes were used to develop dormant (D) and nondormant (ND) pools, each consisting 20 individuals, that is 20 nondormant (germination within 48 hrs) and 20 dormant (FSD up to 13–15; Figure S1 and Table S2) RILs. The phenotypic variability present in RIL population was used for developing two pools with extreme phenotypes that is dormant and nondormant (Figure 1c).

### Whole‐genome sequencing and SNP identification

Sequencing data were generated for the dormant parent (ICGV 97045), the dormant (D pool) and nondormant pool (ND pool). A total of 264.2 million reads for dormant parent (ICGV 97045), 215.5 million reads for dormant (D pool) and 223.7 million reads for nondormant (ND pool) were generated (Tables 1 and S3). Highest amount of sequencing data was generated for the dormant parent ICGV 97045 (33.03 Gb), followed by ND pool (27.97 Gb) and D pool (26.94 Gb). After filtering, the dormant parent ICGV 97045 (200.5 millions) had the maximum high‐quality reads as compared to ND pool (157.4 million) and D pool (156.9 million). The alignment of reads generated for the dormant genotype (ICGV 97045) achieved 85.79% genome coverage and 8.02X of average read depth and resulted in development of reference‐guided based assembly for ICGV 97045 (hereafter referred as ICGV 97045 assembly; Figures S1 and S2). In the FSD dormant pool, the ICGV 97045 assembly resulted in 85.30% coverage and 6.49X read depth, while nondormant to the ICGV 97045 assembly resulted in 85.12% coverage and 6.50X read depth (Tables 1 and S3). SNPs were identified based on the SNP index calculation by comparing each pool to dormant parent, ICGV 97045 (Figures S2, S3 and S4). SNP index corresponds to the frequency of each parental allele in the population of pooled samples. For instance, 0.5 SNP index indicates equal contribution of alleles from both parents (Figures S3 and S4). Therefore, if the SNP is linked to FSD trait, the SNP index for that SNP site associated with D pool would be >0.5, whereas SNP index <0.5 in the ND pool. In general, greater absolute value of ∆SNP index indicates higher probability for the association of SNP site with trait. Thus, to identify key genomic regions and genes associated with FSD trait, we analysed genomewide SNP index with a sliding window of 2‐Mb interval with an increment of 50 kb for D and ND pools which deviated from allele frequency of 0.5. After SNP index calculation, ΔSNP was calculated with a minimum statistical confidence of *P* < 0.05 (Figure S5; Table S4). Thus, after examining the dormant and nondormant pools, a total of 10,759 genomewide SNPs for FSD were identified (Table S4). Of the 10,759 SNPs, A‐ and B‐genome harboured 5,970 and 5,184 SNPs (Tables 2, 3 and S4), respectively. Altogether, 5,452 SNPs were found to be intergenic, 312 SNPs intronic, 90 SNPs synonymous, 178 SNPs nonsynonymous, eight SNPs resulted in stop codon, seven SNPs at spice junctions, 29 SNPs in 3ʹUTR and 13 SNPs in 5ʹ UTR (Tables 2 and 3).

**Table 1:** Summary of Illumina sequencing of parental lines and pools for fresh seed dormancy

Sample ID	Genotype/pools	Total data generated (Gb)	% Alignment	Genome coverage (%)	Average depth (X)
Dormant parent	ICGV 97045	33.03	87.8	85.79	8.02
Dormant pool	D pool	26.94	95.1	85.30	6.49
Nondormant pool	ND pool	27.96	95.2	85.12	6.50

**Table 2:** Genomewide distribution of SNPs and their categories identified in A‐subgenome

Chromosome	Length (Mbp)	Total SNPs	Significant SNPs (∆SNP −1 or 1)	SNPs categories	Total SNPs	Significant SNPs (∆SNP −1 or 1)
Araip.A01	107.00	315	26	Intergenic	5455	710
Araip.A02	93.87	539	70	Intron	311	35
Araip.A03	135.10	203	14	Intron splice junction	2	0
Araip.A04	123.60	332	25	Synonymous	52	6
Araip.A05	110.00	195	14	Non‐synonymous_Missense	105	14
Araip.A06	112.80	298	11	Non‐synonymous_Stop gained	4	0
Araip.A07	79.13	136	8	UTR_3_PRIME	28	2
Araip.A08	49.46	204	22	UTR_5_PRIME	13	0
Araip.A09	120.70	1993	221	**Total**	**5970**	**767**
Araip.A10	109.50	1755	356			
**Total**	**1041.16**	**5970**	**767**			

**Table 3:** Genomewide distribution of SNPs and their categories identified in B‐subgenome and their effect

Chromosome	Length (Mbp)	Total SNPs	Significant SNPs (∆SNP −1 or 1)	SNPs categories	Total SNPs	Significant SNPs (∆SNP −1 or 1)
Araip.B01	137.40	282	13	Intergenic	4789	1023
Araip.B02	109.00	229	12	Intron	246	36
Araip.B03	136.10	153	10	Intron splice junction	5	0
Araip.B04	133.60	348	44	Synonymous	38	3
Araip.B05	149.9	2714	897	Non‐synonymous_Missense	73	7
Araip.B06	137.10	347	28	Non‐synonymous_Stop gained	4	0
Araip.B07	126.40	295	29	UTR_3_PRIME	16	2
Araip.B08	129.60	199	11	UTR_5_PRIME	13	0
Araip.B09	147.10	362	17	**Total**	**5184**	**1071**
Araip.B10	136.20	255	10			
**Total**	**1342.40**	**5184**	**1071**			

### Identification of genomic regions and candidate genes for fresh seed dormancy

**Figure 2:**
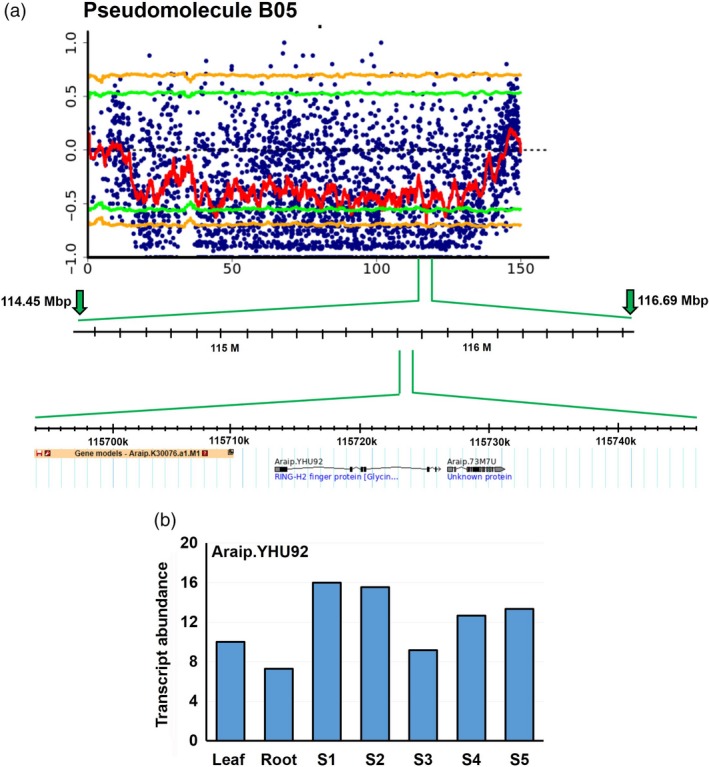
Details of QTL region identified on the pseudomolecule B05 and expression of gene *Araip*.*YHU92*. (a) A closer view of the genomic region identified for FSD (114.45–116.69 Mb) harbouring SNP on gene *Araip*.*YHU92*; (b) transcript abundance of gene *Araip.YHU92*. Stages S1 to S5 represent successive stages of seed development in chronological order.

**Figure 3:**
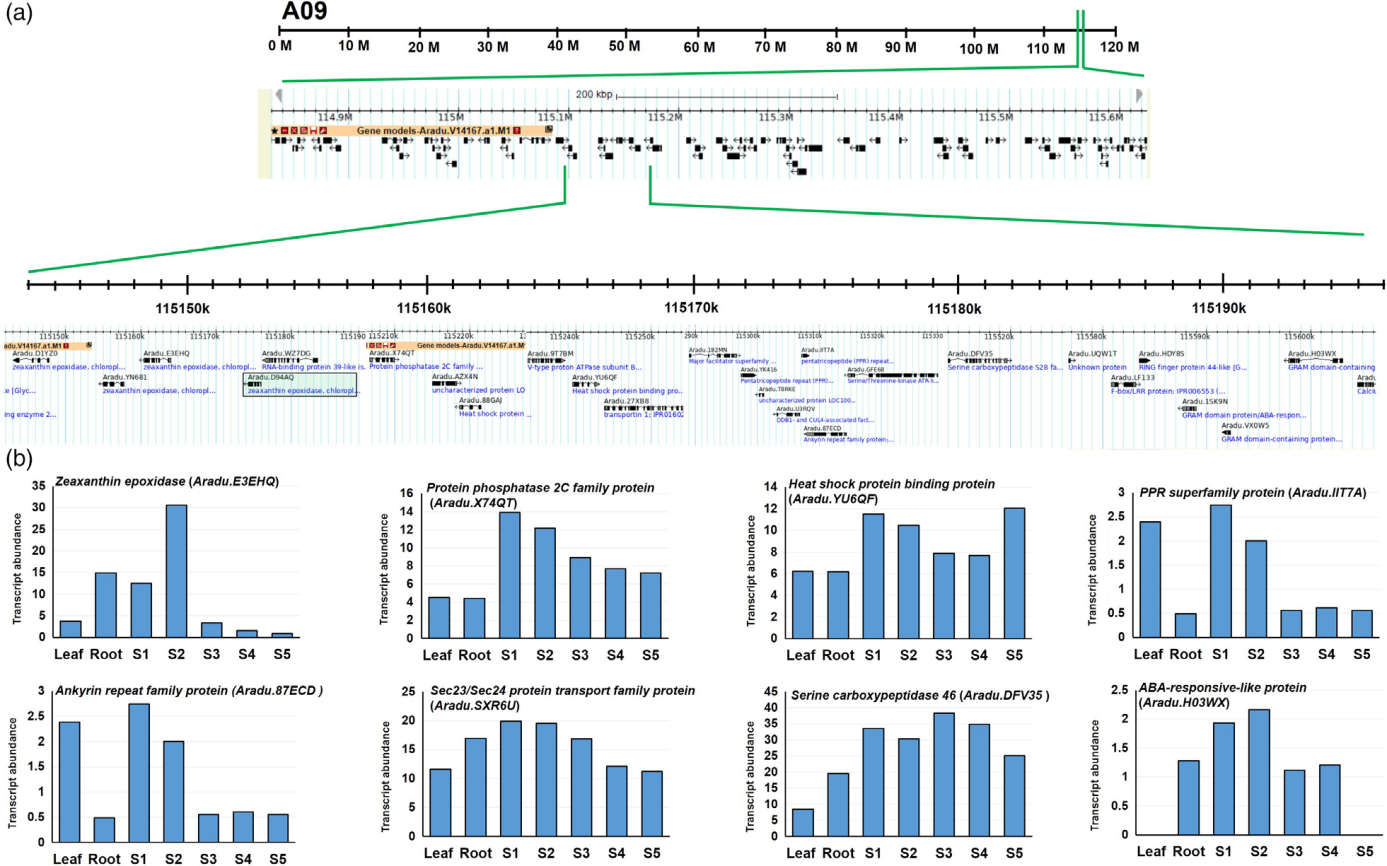
Detail of genomic region identified on the pseudomolecule A09. (a) A closer view of a region harbouring nonsynonymous SNP on ABA biosynthetic gene *zeaxanthin epoxidase* (*Aradu.D94AQ*) and its other three homologs *Aradu.D1YZ0, Aradu*.*YN681* and *Aradu.E3EhQ*. Black box indicates gene harbouring nonsynonymous SNP. (b) Transcript abundance of some of the important genes which are present this genomic region.

After computing SNP index and ΔSNP index in the two extreme pools D and ND, a FSD‐associated genomic region was identified on the pseudomolecule B05 from 114.45 Mb to 116.69 Mb (2.24 Mb) of B‐subgenome (Figure 1). This genomic region harboured total 52 SNPs (Table 4), and 18 of these SNPs were highly significant (*P* value ≤ 0.01, ΔSNP index = −1). The negative sign of ΔSNP index indicates the presence of biasedness in the inheritance of parental genomes in the pools towards dormant parent or *vice versa*. The dormant pool had SNP index = 0 at all SNP positions indicating the contribution of alleles coming from the dormant parent, ICGV 97045 (Table 4). Similarly, the nondormant pool scored SNP index = 1 indicating the source of alleles for nondormancy from nondormant parent (ICGV 00350). Of the 52 SNPs, 49 SNPs were intergenic and three were intronic affecting three genes *viz Araip.S6QRU* (encodes receptor‐like protein kinase), *Araip.YHU92* (encodes RING‐H2 finger protein; Figure 2) and *Araip.X9V0W* (encodes SOUL heme‐binding family protein; Table 4). Previously, role of RING‐H2 finger protein has been demonstrated during ABA biosynthesis and signalling (Bu *et al*., 2009). Notably, Clevenger *et al*. (2016) has reported significantly high expression of gene *Araip.YHU92* during seed development (Figure 3; https://peanutbase.org/search/gene), especially at the later stages of maturity when endogenous ABA accumulates (Rodríguez‐Gacio *et al*., 2009), suggesting its plausible role during seed development and ABA metabolism in groundnut. Additionally, this genomic region consists of 46 important genes which control the growth and development of seed as well as dormancy (Table S5A).

**Table 4:** List of SNPs identified in the QTL region pseudomolecule B05 and their significance

Chromosome	Position	Reference call	Alternate call	Delta SNP index	U99 (99% confidence)	L99 (99% confidence)	Gene ID	Effect type	Gene description
Araip.B05	114451556	T	A	−1	0.800	−0.800		INTERGENIC	
Araip.B05	114543183	G	A	−0.86	0.800	−0.800		INTERGENIC	
Araip.B05	114643837	G	T	0.63	0.800	−0.800		INTERGENIC	
Araip.B05	114729123	G	A	−0.62	0.700	−0.700		INTERGENIC	
Araip.B05	114759444	T	A	−0.83	0.833	−0.833		INTERGENIC	
Araip.B05	114768155	G	A	−0.83	0.833	−0.833		INTERGENIC	
Araip.B05	114778599	C	A	−0.75	0.750	−0.750		INTERGENIC	
Araip.B05	114796399	C	T	−0.78	0.666	−0.666		INTERGENIC	
Araip.B05	114864254	G	A	−0.88	0.833	−0.833		INTERGENIC	
Araip.B05	114889476	G	A	−0.83	0.800	−0.800		INTERGENIC	
Araip.B05	114893409	G	T	−0.86	0.800	−0.800		INTERGENIC	
Araip.B05	114947595	G	A	−1	0.714	−0.714		INTERGENIC	
Araip.B05	115014514	A	G	−0.67	0.833	−0.833		INTERGENIC	
Araip.B05	115077058	A	G	−1	0.800	−0.800		INTERGENIC	
Araip.B05	115081834	G	C	−0.86	0.833	−0.833		INTERGENIC	
Araip.B05	115084232	T	G	−0.75	0.750	−0.750	Araip.S6QRU	INTRON	probable receptor‐like protein kinase
Araip.B05	115182881	G	T	−0.83	0.833	−0.833		INTERGENIC	
Araip.B05	115373942	G	A	−1	0.800	−0.800		INTERGENIC	
Araip.B05	115375662	G	T	−1	0.833	−0.833		INTERGENIC	
Araip.B05	115544050	G	A	−0.9	0.833	−0.833		INTERGENIC	
Araip.B05	115568667	C	T	−1	0.800	−0.800		INTERGENIC	
Araip.B05	115578247	C	A	−0.80	0.800	−0.800		INTERGENIC	
Araip.B05	115606146	T	C	−0.75	0.750	−0.750		INTERGENIC	
Araip.B05	115621197	G	A	−0.90	0.833	−0.833		INTERGENIC	
Araip.B05	115622058	C	A	−0.57	0.700	−0.700		INTERGENIC	
Araip.B05	115631384	C	T	−1	0.800	−0.800		INTERGENIC	
Araip.B05	115634902	G	A	−0.86	0.714	−0.714		INTERGENIC	
Araip.B05	115697798	C	T	−1	0.800	−0.800		INTERGENIC	
Araip.B05	115717881	G	A	−1	0.700	−0.700	Araip.YHU92	INTRON	RING‐H2 finger protein
Araip.B05	115753322	G	A	−0.83	0.636	−0.636		INTERGENIC	
Araip.B05	115754681	T	C	−0.88	0.750	−0.750		INTERGENIC	
Araip.B05	115878234	C	T	−0.71	0.800	−0.800		INTERGENIC	
Araip.B05	115972726	C	T	−1	0.714	−0.714		INTERGENIC	
Araip.B05	115998546	G	A	−0.73	0.800	−0.800		INTERGENIC	
Araip.B05	116010768	G	A	−0.91	0.750	−0.750		INTERGENIC	
Araip.B05	116022147	G	T	−0.78	0.750	−0.750		INTERGENIC	
Araip.B05	116061110	G	A	−1	0.833	−0.833		INTERGENIC	
Araip.B05	116085057	G	T	−1	0.666	−0.666		INTERGENIC	
Araip.B05	116143047	G	T	−0.80	0.800	−0.800		INTERGENIC	
Araip.B05	116143048	A	G	−0.80	0.800	−0.800		INTERGENIC	
Araip.B05	116155115	G	A	−0.83	0.800	−0.800		INTERGENIC	
Araip.B05	116160728	C	A	−1	0.833	−0.833		INTERGENIC	
Araip.B05	116198871	C	T	−0.75	0.750	−0.750		INTERGENIC	
Araip.B05	116205168	G	A	−0.76	0.750	−0.750		INTERGENIC	
Araip.B05	116215363	G	A	−0.82	0.833	−0.833		INTERGENIC	
Araip.B05	116237485	C	G	−1	0.714	−0.714		INTERGENIC	
Araip.B05	116426085	T	A	−1	0.714	−0.714		INTERGENIC	
Araip.B05	116563656	G	A	−1	0.800	−0.800		INTERGENIC	
Araip.B05	116581795	G	T	−0.75	0.800	−0.800		INTERGENIC	
Araip.B05	116670829	G	T	−1	0.714	−0.714	Araip.X9V0W	INTRON	SOUL heme‐binding family protein
Araip.B05	116676958	C	T	−0.88	0.833	−0.833		INTERGENIC	
Araip.B05	116695578	G	C	−1	0.800	−0.800		INTERGENIC	

Notably, during analysis of WGS data, we computed ΔSNP index to identify nonsynonymous SNPs distributed on 20 pseudomolecules. During analysis, we observed a nonsynonymous SNP (with ΔSNP index = −1) on one gene *Aradu.D94AQ*, which encodes for an abscisic acid biosynthetic enzyme zeaxanthin epoxidase. This gene was located in the genomic region of A09 pseudomolecule (114873000..115603500; 0.74 Mb) of A‐genome which is appeared to be a hot spot for genes regulating seed development and ABA signalling (Figure 3, Table S5). This genomic region consists of 24 important genes *viz.,* four homologs of *zeaxanthin epoxidase* (*Aradu.D94AQ, Aradu.D1YZ0, Aradu.YN681* and *Aradu.E3EHQ*), seven homologs of *pentatricopeptide repeat* (PPR) superfamily protein genes (*Aradu.S2S1T, Aradu.YK416, Aradu.IIT7A, Aradu.D14WA, Aradu.GPN3U, Aradu.64B2V, Aradu.61TTJ* and *Aradu.P7UBS*), two homologs of protein *phosphatase 2C/2A family protein* (*Aradu.X74QT* and *Aradu.NF928*), three homologs of *GRAM domain protein/ABA‐responsive‐like* (*Aradu.1SK9N, Aradu.VX0W5* and *Aradu.H03WX*), two homologs of *heat shock proteins family* genes (*Aradu.YU6QF* and *Aradu.88GAJ*) and one homolog for each *serine carboxypeptidase 46* (*Aradu.DFV35*), *Sec23/Sec24 protein transport family protein* (*Aradu.SXR6U*), *Ankyrin repeat family protein* (*Aradu.87ECD*) and *DDB1‐ and CUL4‐associated factor 8‐like* (*Aradu.U3RQV*). Previously, these genes have been characterized to play important role in ABA biosynthesis/signalling, seed dormancy and germination (Table S6). As the developmental transcriptome map for cultivated groundnut species is available in public domain (Clevenger *et al*., 2018; https://peanutbase.org/search/gene/), it provides the complete picture of transcriptomic shift for 22 different tissue including five important developmental stages of seed. Therefore, we surveyed the transcript abundance of these genes in the seed at different developmental stages. Interestingly, we found many of them identified as seed‐specific or highly expressed during development of groundnut seeds (Figure 3; Table S5B).

### Development and validation of allele‐specific markers for fresh seed dormancy

**Figure 4:**
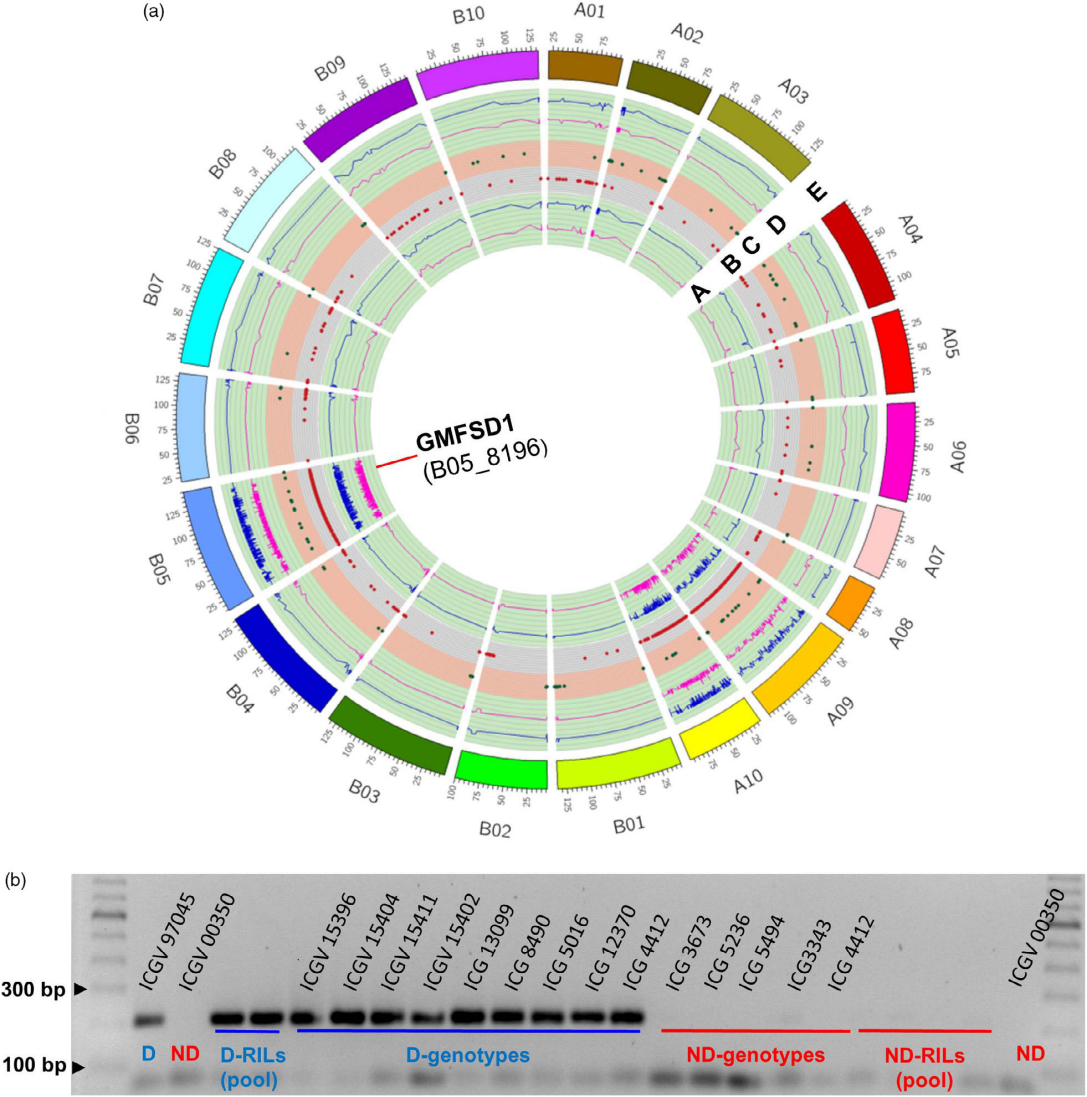
Validation of a linked marker. (a) A circos map representing GMFSD1 (B05_8196) marker developed from the B‐subgenome. A. Lower probability values at 99% confidence (*P* < 0.01) and 95% confidence (*P* < 0.05) for declaring Δ index and a marker GMFSD1 identified on chromosome B05; B. genomewide ΔSNP index = 1 and contributed by dormant parent (ICGV 97045); C. genomewide ΔSNP index = −1 and contributed by non‐dormant parent (ICGV 00350); D. upper probability values at 99% confidence (*P* < 0.01) and 95% confidence (*P* < 0.05) for declaring significant ΔSNP index; E. 20 chromosomes of *Arachis hypogaea* with their respective start and end positions. (b) Marker validated on a validation panel comprising a set of parents, RIL bulks (D and ND) and breeding material.

Based on the ΔSNP index, a total of 143 SNPs (located in QTL region and other pseudomolecules; see Table S7) were targeted for the development of allele‐specific markers. Of 143 allele‐specific markers, 42 markers showed clear polymorphism between dormant and nondormant parents. These 42 polymorphic markers were tested on D and ND pool of RILs and a set of breeding material to identify the promising marker(s) which co‐segregate with the dormancy phenotype (Table S6). As a result, of these 42 markers, one marker GMFSD1 (B05_8196) showed consistency in differentiating the parents, pooled samples as well as a set of breeding material for dormancy and nondormancy (Figure 4).

## Discussion

Advances in the field of genomics have brought a dramatic reduction in the cost of sequencing technologies. Advent of next‐generation sequencing (NGS) technologies has greatly facilitated development of genome assembly, trait mapping and candidate gene discovery (Varshney *et al*., 2015). Thus, NGS technology has augmented faster and precise trait discovery through rapid detection of linked genomic regions (also gene discovery), trait‐associated polymorphism and identification of diagnostic markers (Varshney *et al*., 2015, 2018a,b). There are several sequencing‐based trait‐mapping approaches which provide faster discovery of candidate genes and facilitate marker development; and one of such approach for trait mapping is QTL‐seq (Pandey *et al*., 2017; Takagi *et al*., 2013). It has been successfully deployed in several important crop species for trait dissection such as rice blast disease (Takagi *et al*., 2013), grain length and weight in rice (Yaobin *et al*., 2018), flowering time in tomato (Ruangrak *et al*., 2018), foliar disease resistance (Clevenger *et al*., 2018; Pandey *et al*., 2017), shelling percentage (Luo *et al*., 2019a) and bacterial wilt resistance (Luo *et al*., 2019b) in groundnut, 100 seed weight and root/total plant dry weight in chickpea (Singh *et al*., 2016a), plant height in soybean (Zhang *et al*., 2018), etc. Many of these efforts facilitated successful development of diagnostic markers which are being deployed in GAB. In coming years, there will be a shift from GAB to sequence‐based breeding (Varshney *et al*., 2018a) and these SNP‐based diagnostic markers will be of great use in enhancing the modernization and precision of breeding programmes for achieving higher genetic gains in farmers’ field (Varshney *et al*., 2018b).

FSD/PHST/*in situ* germination leads to a reduction in the grain or kernel yield and often attributes to medium to large yield losses and quality deterioration of the produce in both cereals (Abe et al., 2019; Benech‐Arnold and Rodríguez, 2018; Gao and Ayele, 2014; Nakamura, 2018; Rodríguez *et al*., 2015) and legumes (Dias *et al*., 2011; Patro and Ray, 2016; Vishwakarma *et al*., 2016). Due to domestication and extensive human‐made selection during varietal development programs, modern groundnut represents much low genetic diversity compared to its wild ancestral species (which produces dormant seeds). As a result, *in situ* sprouting of groundnut seeds caused due to lack of FSD results in a loss of up to 20%–50% (Nautiyal *et al*., 2001; Yaw *et al*., 2008). Several researchers have studied this trait in order to find suitable new sources for FSD in groundnut (Faye *et al*., 2009; Patro and Ray, 2016; Yaw *et al*., 2008). Our previous study, the only one, was performed in F_2_ population derived from the cross between ICGV 00350 and ICGV 97045 which provided preliminary idea on inheritance pattern and reported two major QTL regions associated with FSD using Diversity Arrays Technology (DArT) and DArTseq platform (Vishwakarma *et al*., 2016). The DArT and DArTseq are useful genotyping approaches but they do not provide exact position of SNPs in the reference genome which is very much required for performing fine mapping, candidate gene discovery and marker development. Earlier, it has been successfully demonstrated by Sato *et al*. (2016) that the previously reported candidate genes can be validated the previously reported candidate gene, followed by its use in improving target trait. Therefore, we advanced the same population in this study for applying QTL‐seq approach to perform high‐resolution trait mapping and identify genomic region(s) associated with FSD. The use of highly homozygous RIL‐F_7_ line provided highly significant SNPs and genomic region(s) associated with the desired trait for the discovery of candidate genes. As a result, sequence comparison discovered distribution of 5,970 and 5,184 SNPs (*P* value ≤ 0.01) on A‐ and B‐genome; among them only 767 and 1071 SNP were with ∆SNP index equivalent to −1. These highly significant SNPs (*P* value ≤ 0.01, ∆SNP index = −1) were mostly distributed among pseudomolecule—A09 (221 out of 767), A10 (356 out of 767) and B05 (897 out of 1071), suggesting plausible association of these three pseudomolecules with FSD trait.

The comprehensive QTL‐seq analysis for FSD detected a major genomic region of 2.24 Mb on the pseudomolecule B05 of B‐subgenome. This promising region contains 46 important genes, and four of these genes were found with important functions namely *SOUL heme‐binding family protein* (*Araip.X9V0W*), *RING‐H2 finger protein* (*Araip.YHU92*), *far1‐related sequence 6‐like* (*Araip.LKN71*), *ascorbate oxidase* (*Araip.K7Y13*) and *heavy metal transport/detoxification superfamily protein* (*Araip.2F1GS*). Sequencing data suggested synonymous SNPs among genes encoding RING‐H2 finger protein, far1‐related sequence 6‐like and SOUL heme‐binding family protein, which are key regulator of developmental processes (Bu *et al*., 2009; Ko *et al*., 2006; Li *et al*., 2018). Likewise, a 0.74‐Mb SNP‐enriched genomic region was identified on the pseudomolecule A09 of A‐subgenome, containing a nonsynonymous SNP on an ABA biosynthetic gene *zeaxanthin epoxidase* (*ZEP, Aradu.D94AQ*). Further, this genomic appeared as one of the key controllers of ABA biosynthesis in groundnut as this genomic region is home to several important genes which are known to participate in ABA signalling and control seed dormancy such as *PPR superfamily protein* gene, *protein phosphatase 2C/2A family protein* gene, *GRAM domain protein/ABA‐responsive‐like* and *serine carboxypeptidase 46* (Mauri *et al*., 2016; Née *et al*., 2017; Xia *et al*., 2018).

Abscisic acid crosstalk with other hormones such as ethylene and gibberellic acids (GAs) is crucial during plant developmental processes including seed development and germination (Rodríguez‐Gacio *et al*., 2009). It is well‐accepted fact that ABA signalling plays an important role in seed dormancy, affecting seed germination process (Dejonghe *et al*., 2018; Finch‐Savage and Leubner‐Metzger, 2006; Liu *et al*., 2013; Nishimura *et al*., 2018). In Arabidopsis, a transcription factor RING‐H2 finger protein positively regulates the ABA biosynthesis and signalling during seed germination (Bu *et al*., 2009) and confers abiotic stress tolerance through increased ABA biosynthesis (Ko *et al*., 2006; Liu *et al*., 2016). Notably, overexpression of an Arabidopsis RING‐H2 finger protein‐encoding gene, *XERICO*, confers drought tolerance through enhanced ABA accumulation (Ko *et al*., 2006). As anticipated from these previous findings, the expression of gene *RING‐H2 finger protein* (*Araip.YHU92*) is also substantially high in groundnut developing seeds, suggesting its role in ABA accumulation, which might also impact seed dormancy.

Zeaxanthin epoxidase is one of the key enzymes involved in ABA biosynthesis, which catalyses the first step of ABA biosynthesis by epoxidation of zeaxanthin to violaxanthin (Thompson *et al*., 2000). Earlier, role of ZEP in seed dormancy has been demonstrated in Arabidopsis and tobacco (Frey *et al*., 1999; Marin *et al*., 1996) and peach (Wang *et al*., 2016). Also, a QTL analysis for preharvest sprouting in Arabidopsis has identified *DOG* locus (Alonso‐Blanco *et al*., 2003), now identified as one of the key regulators in the ABA signalling pathway (Nishimura *et al*., 2018). Further, in rice, ABA receptor mutants showed pleotropic effects including seed dormancy and rice productivity (Miao *et al*., 2018). Further, dormancy and germination are complex physiological process involving complex interaction between several pathways, including redox signalling through reactive oxygen species (ROS). We also identified a ROS‐scavenging enzyme ascorbate oxidase encoding gene *Araip.K7Y13* in the genomic region identified on B05. In practical terms, our data suggest several candidate genes controlling FSD in groundnut. Among them, *ZEP* (*Aradu.D94AQ*) and *RING‐H2 finger protein* (*Araip.YHU92*) might be the most relevant genes regulating preharvest sprouting caused due to lack of FSD in ground nut. Further, it would be interesting to know the genetic regulation of *RING‐H2 finger protein* transcription factor over ABA synthesis, as it could be plausible that this transcription factor might be binding to the promoter region of *ZEP* and ABA biosynthetic genes including *NCED*. Additionally, finding haplotypes associated with SNPs in these genes in the natural population(s) and breeding material and characterization of these gene(s) by genome editing tools will provide new insight towards the genetic control of FSD.

Major advantage of sequencing based trait mapping approaches is mapping accurate genomic regions on the genome, discovery of candidate genes in addition to development of DNA markers. Earlier, Pandey *et al*. (2017) and Luo *et al*. (2019a and) Luo *et al*. (2019b) have demonstrated the benefits of QTL‐seq approach; identification of candidate gene(s) and development of markers in the candidate genomic region. The present study also successfully developed a marker GMFSD1 near the identified genomic region on the B05 which is closely linked to the one of the candidate gene *Araip.YHU92*. This marker was able to distinguish both parents and the extreme bulks and successfully differentiated both diverse breeding and germplasm materials with FSD trait. Thus, our study provides an important marker linked to FSD, which can be used in groundnut breeding programme for early section of FSD trait.

In summary, our study suggests WGS‐based QTL‐seq approach as one of the most efficient techniques for the identification putative regions/SNPs associated with desired traits. Further, it is reasonable to target *ZEP* and *RING‐H2 finger protein* as candidate genes for introgression of fresh seed dormancy. Furthermore, more evidences are required to functionally validate these genes; plausibly genome editing would be best to deliver this. Therefore, we are interested to characterize the impact of candidate genes identified in the present study through overexpression study as well as CRISPR/Cas9. Additionally, we have developed multiple RIL populations which will be helpful in delineating and identifying additional candidate genes and markers for use in marker‐assisted breeding to improve FSD in groundnut.

## Materials and methods

### Plant materials and construction of pools based on phenotyping

The RIL population derived from the cross ICGV 00350 × ICGV 97045, earlier described by Vishwakarma *et al*. (2016), was used in this study. Both parents used in this study are short‐duration Spanish varieties (subspecies *fastigiata*; botanical type *vulgaris*) and are widely cultivated in the states of Tamil Nadu and Andhra Pradesh in India under both irrigated and rain‐fed condition. The nondormant parent, ICGV 00350, is drought‐tolerant genotype, but prone to sprout in the field, that is lacks fresh seed dormancy. In contrast, ICGV 97045 possesses fresh seed dormancy up to 15 days, and being used as donor parent for improving FSD in several varieties at ICRISAT. Our previous genetic mapping effort for FSD used a subset F_2:3_ population (368) (Vishwakarma *et al*., 2016) of large segregating F_2:3_ population (>800). We followed then SSD for rest of the F_2_ lines and finally developed RIL population with 366 lines. For phenotyping, the noncured seed from freshly harvested mature pods (post rainy season) was used for the germination assay and enough care was taken to avoid test a damage while removing seeds from the pods. The maturity of pods was determined by the development of black coloration inside the shell as explained by Miller and Burns (1971). To minimize the experimental variation, good‐quality uniform‐sized 20 seeds from each RIL were chosen for germination assay and the experiment was conducted in technical replicates. The seeds were treated with fungicide Captan, n‐[(trichloromethyl) thio]‐4‐cyclohexene‐l,2‐dicarboxymide, at 2 g/kg seed (Upadhyaya and Nigam 1999), placed on filter paper in a petri dish which was kept moist with sterilized distilled water during the course of the study and were incubated at 35 ± 2 °C in the dark in an incubator, and the seed germination was recorded daily. The dormant RILs which showed FSD upto 13–15 days were used for creating dormant pool/bulk; and the RILs which showed phenotype similar as ICGV 00350 (germinated within 24 h) were used for the nondormant pool/bulk. Based on above phenotyping results, equimolar concentration DNA (100 ng each) of 20 RIL individuals with above‐mentioned extreme phenotype, that is dormant and nondormant seeds, was pooled together to create dormant (D) and nondormant (ND) pool, respectively (Figures 1, S1).

### Sequence libraries construction and Illumina sequencing

The WGS data were generated for three samples namely ICGV 97045 (dormant parent), dormant pool for fresh seed dormancy (D pool) and nondormant pool for non‐fresh seed dormancy (ND pool) were prepared and used for sequencing on Illumina HiSeq 2500 at Center of Excellence in Genomics and Systems Biology (CEGSB), ICRISAT, Hyderabad, as described in Pandey *et al*. (2017). In brief, single Illumina library for each sample was made using TruSeq DNA Sample Prep kit LT (set A) FC‐121‐. Two micrograms DNA from each of these three samples was first sheared using diagenode Bioruptor^®^ NGS and then was subjected to end repairing and adapter ligation. For size selection, 2% agarose gel was used for electrophoresis and 500–600 bp insert size was selected, purified and then enriched by using adaptor compatible PCR primers. The size of the DNA libraries was reconfirmed through chip assay using Agilent Technologies 2100 Bioanalyzer (Agilent Technologies, Palo Alto, CA). Later, these libraries were used to generate 250 bases pair‐end reads by sequencing them on Illumina HiSeq platform with Reagent Kit v2 (500‐cycles).

### Construction of reference‐guided assembly

SNP index was calculated by using QTL‐seq pipeline (http://genome-e.ibrc.or.jp/home/bioinformatics-team/mutmap), developed at Iwate Biotechnology Research Center, Japan. For analysis, a reference tetraploid genome assembly for groundnut was constructed by using diploid genome assemblies of *A. duranensis* (A‐genome) and *A. ipaensis* (B‐genome) developed by Bertioli *et al*. (2016). The cleaned reads of ICGV 97045 were first aligned to the constructed reference tetraploid genome assembly using inbuilt Burrows‐Wheeler Alignertool (Li and Durbin, 2009). Thereafter, we used Coval software for postprocessing and filtering of the alignment files which were developed after aligning sequence reads to both diploid genomes separately (Kosugi *et al*., 2013). Followed by variants call between ICGV 97045 (dormant parent) and the both diploid reference genomes. ICGV 97045 reference‐guided assembly was developed by using these variants and the synthetic tetraploid genome assembly by substituting the alternate bases with high‐confidence SNP variants. Thereafter, reads of both dormant and nondormant pools were then aligned onto ICGV 97045 assembly. The variants (SNP index) were then called for both pool samples with ICGV 97045 assembly.

### Implementation of QTL‐seq pipeline and SNP index calculation

SNP index for both pools was calculated by equating with the ICGV 97045 assembly using a formula previously described method (Abe *et al*., 2012; Pandey *et al*., 2017; Takagi *et al*., 2013). SNP index at a position in a pseudomolecule is derived by division of the counts of alternate base with the number of reads aligned. The SNP positions with read depth <5 in both the pools and SNP index <0.3 in either of the pool were filtered out. ∆SNP index was then calculated by subtracting SNP index of nondormant pool from SNP index of dormant pool. It is important to mention that only those SNPs were selected for ∆SNP index calculation that had homozygous alleles in both pools. SNPs which passed the criteria of having ∆SNP index = −1 were considered as causal SNPs for FSD. ∆SNP index = −1 indicates that the allele called in dormant pool was same as that of dormant parent, while alternate base in nondormant pool or *vice versa* (Figure S6). These ∆SNP index value was used for QTLs identifications. In order to find minor alleles or important SNPs controlling FSD traits, we also analysed the overall ∆SNP index data obtained through whole‐genome resequencing data by comparing SNP index of nondormant pool from SNP index of dormant pool to find the SNPs associated with important genes which are known to be involved in seed development and hormone homeostatsis including ABA signalling and synthesis.

### Designing of primer pairs, polymerase chain reaction (PCR)

Based on the ∆SNP index, candidate SNPs were targeted to design allele‐specific primers (marker) using Primer3 (http://primer3.ut.ee/; You *et al*., 2008; Table S7). To avoid complication and have PCR condition consistency, we specifically designed allele‐specific primer pairs with Tm of 61–62 °C.

Polymerase chain reaction was carried out according to a previous report with minor modifications (Pandey *et al*., 2017). In brief, the PCR mix of 15 μL consisted of 5 ng of DNA template, 1X PCR buffer, 2.5 mm each dNTPs, 2.0 mm MgCl_2_, 0.12 μL Taq polymerase (KAPA Biosystems) and 3 pmoles (0.15 μm) each of forward and reverse primers. The cycling conditions for PCR amplification were 94 °C‐4 min, 5 cycles of 94 °C‐20 s, 62 °C‐30 s, 72 °C‐30 s (extension), followed by 30–35 cycles of 94 °C‐20 s, 58 °C‐30 s, 72 °C‐30 s, final extension at 72 °C‐10 min.

After PCR amplification, the alleles were scored on 2% agarose gel electrophoresis as present and absent. The markers were amplified using both dormant and nondormant parent parents used for development RIL population. Identified polymorphic markers were used to screen polymorphism between RILs F_7_ individuals (with extreme phenotype) that were used to create dormant and nondormant pool. Identified polymorphic markers were validated in a diverse panel consist of both FSD and non‐FSD accessions for their broader applicability.

## Conflicts of interest

The authors declare no conflict of interest.

## Author contribution

RKV, MKP and PJ conceived the idea. PJ, SSM and MTV developed and phenotyped RIL population. RK, MKV, MKP, YS and SSG performed the experiments. AWK performed the bioinformatics analysis. RK drafted the manuscript, and RKV and MKP finalized the MS.

## Supporting information


**Figure S1** Schematic representation of QTL‐seq approach used for trait mapping in groundnut for fresh seed dormancy.
**Figure S2** Sequencing depth of the dormant parent ICGV 97045 Black line indicates the sliding window average of 2 Mb interval with 50 kb increment for SNP‐index.
**Figure S3** SNP‐index plots for 20 pseudomolecules dormant (D) pool with the dormant parent. Red lines indicate the sliding window average of 2 Mb interval with 50 kb increment for SNP‐index.
**Figure S4** SNP‐index plots for 20 pseudomolecules nondormant (ND) pool with the dormant parent.
**Figure S5** The Δ (SNP index) plot obtained by subtraction of dormant (D) pool SNP‐index from nondormant pool SNP‐index.

**Table S1** FSD phenotyping details of RIL population**.**

**Table S2** Details on the recombinant inbred lines selected for pooling and whole genome re‐sequencing.

**Table S3** Details on whole genome re‐sequencing data generated on parental genotypes and pooled samples using Illumina 2500.

**Table S4** SNP distribution on A‐ and B‐genome.

**Table S5** List of important genes found on the QTL region on pseudomolecule B05 and hot spot region (114850050..115351249) of pseudomolecule A09 and their expression in groundnut seed.

**Table S6** List of genes identified on the A09 hot spot region involved in the ABA biosynthesis and metabolism.

**Table S7** List of selected markers designed and used for SNP validation assay.
