# Supplementary material for: Whole‐genome resequencing‐based QTL‐seq identified candidate genes and molecular markers for fresh seed dormancy in groundnut
Source: Plant Biotechnol J. 2019 Oct 20;18(4):992–1003. doi: 10.1111/pbi.13266 (PMC7061874; doi:10.1111/pbi.13266)
Supplement: Supplementary file 1 — Figure S1 Schematic representation of QTL‐seq approach used for trait mapping in groundnut for fresh seed dormancy. Figure S2 Sequencing depth of the dormant parent ICGV 97045 Black line indicates the sliding window average of 2 Mb interval with 50 kb increment for SNP‐index. Figure S3 SNP‐index plots for 20 pseudomolecules dormant (D) pool with the dormant parent. Red lines indicate the sliding window average of 2 Mb interval with 50 kb increment for SNP‐index. Figure S4 SNP‐index plots for 20 pseudomolecules nondormant (ND) pool with the dormant parent. Figure S5 The Δ (SNP index) plot obtained by subtraction of dormant (D) pool SNP‐index from nondormant pool SNP‐index. [file PBI-18-992-s005.pdf]

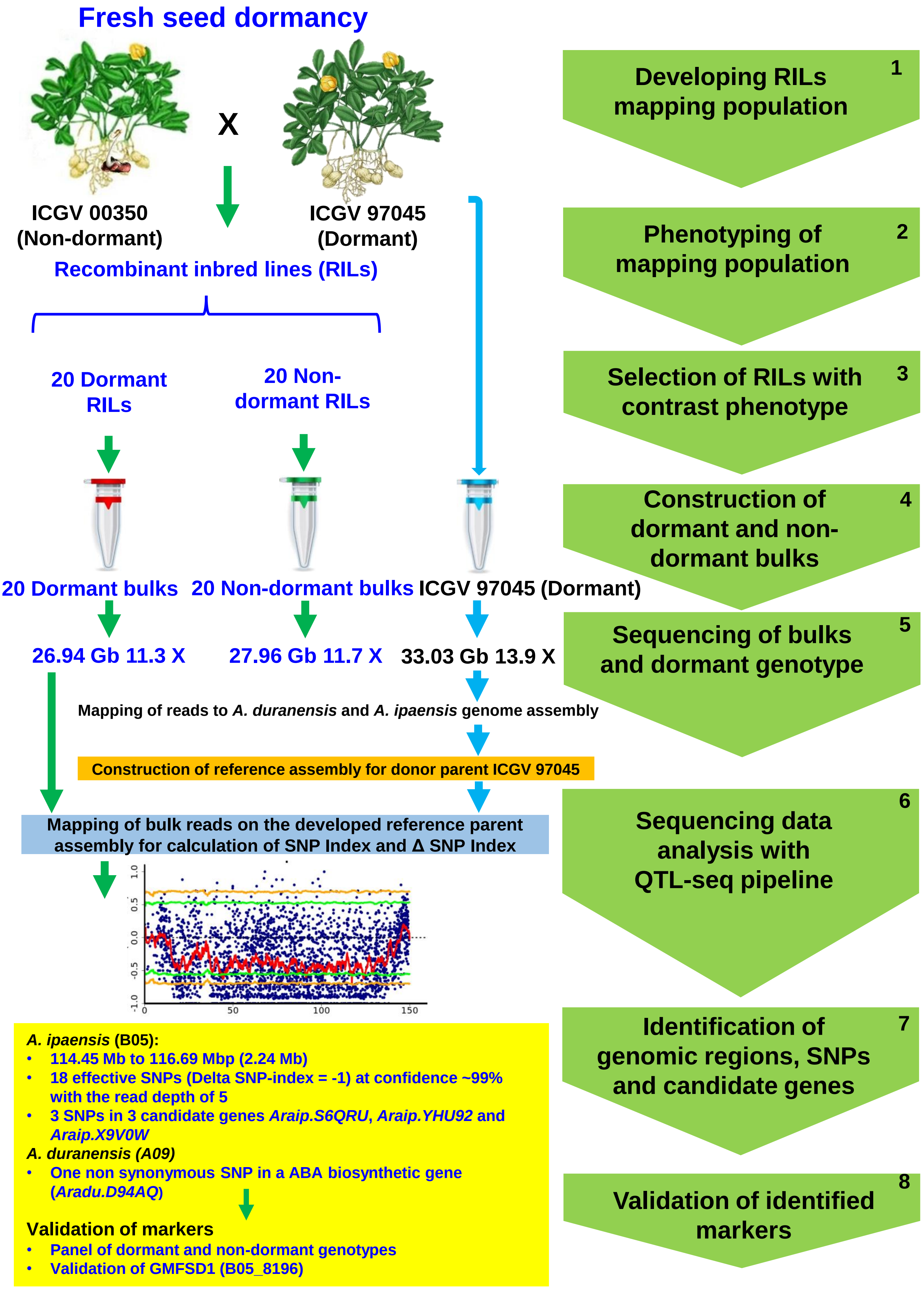

Supplementary Figure S1. QTL-seq approach used for trait mapping in groundnut for fresh seed dormancy

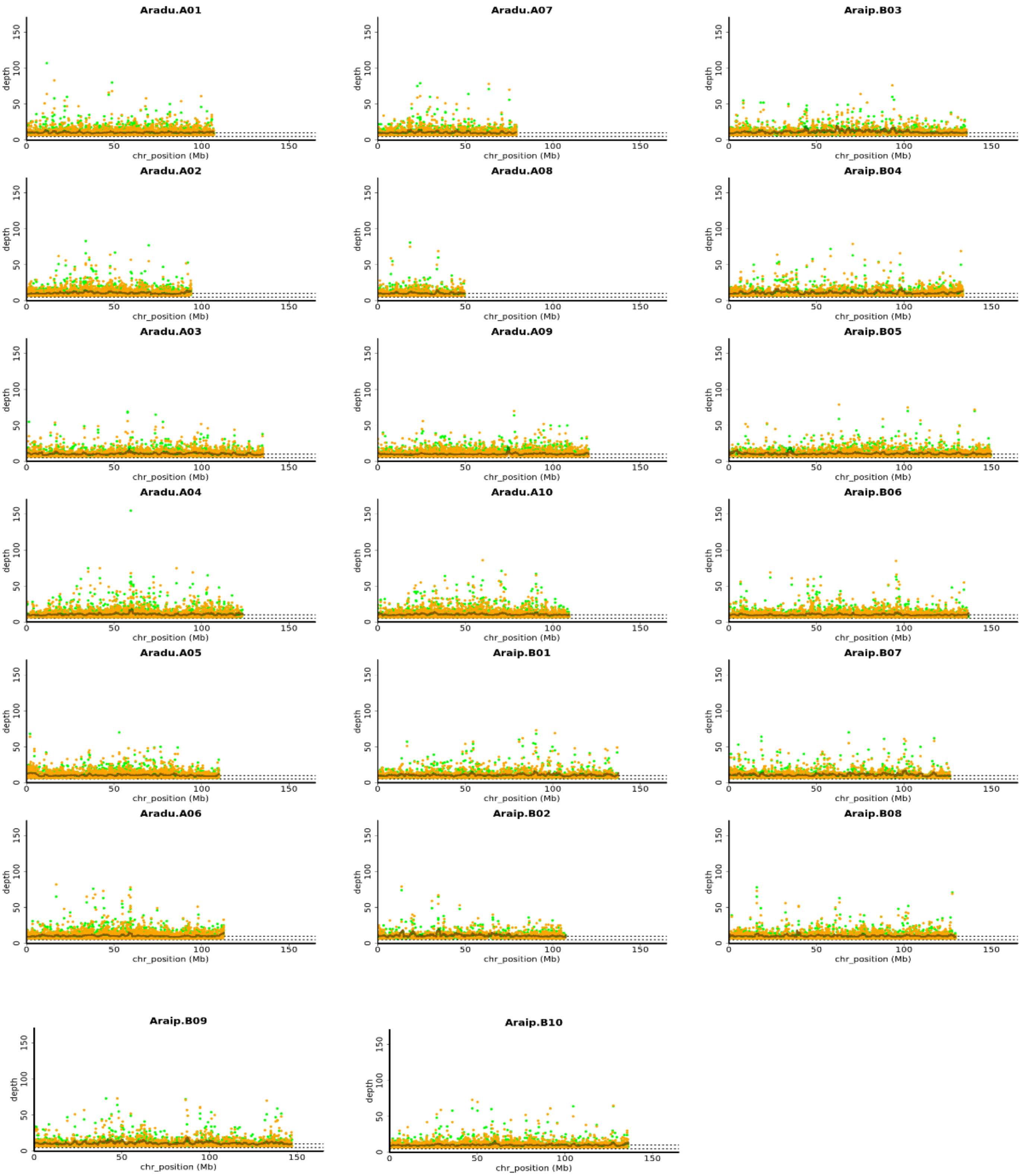

Supplementary Figure S2. Sequencing depth of the dormant parent ICGV 97045 Black line indicates the sliding window average of 2 Mb interval with 50 kb increment for SNP-index.

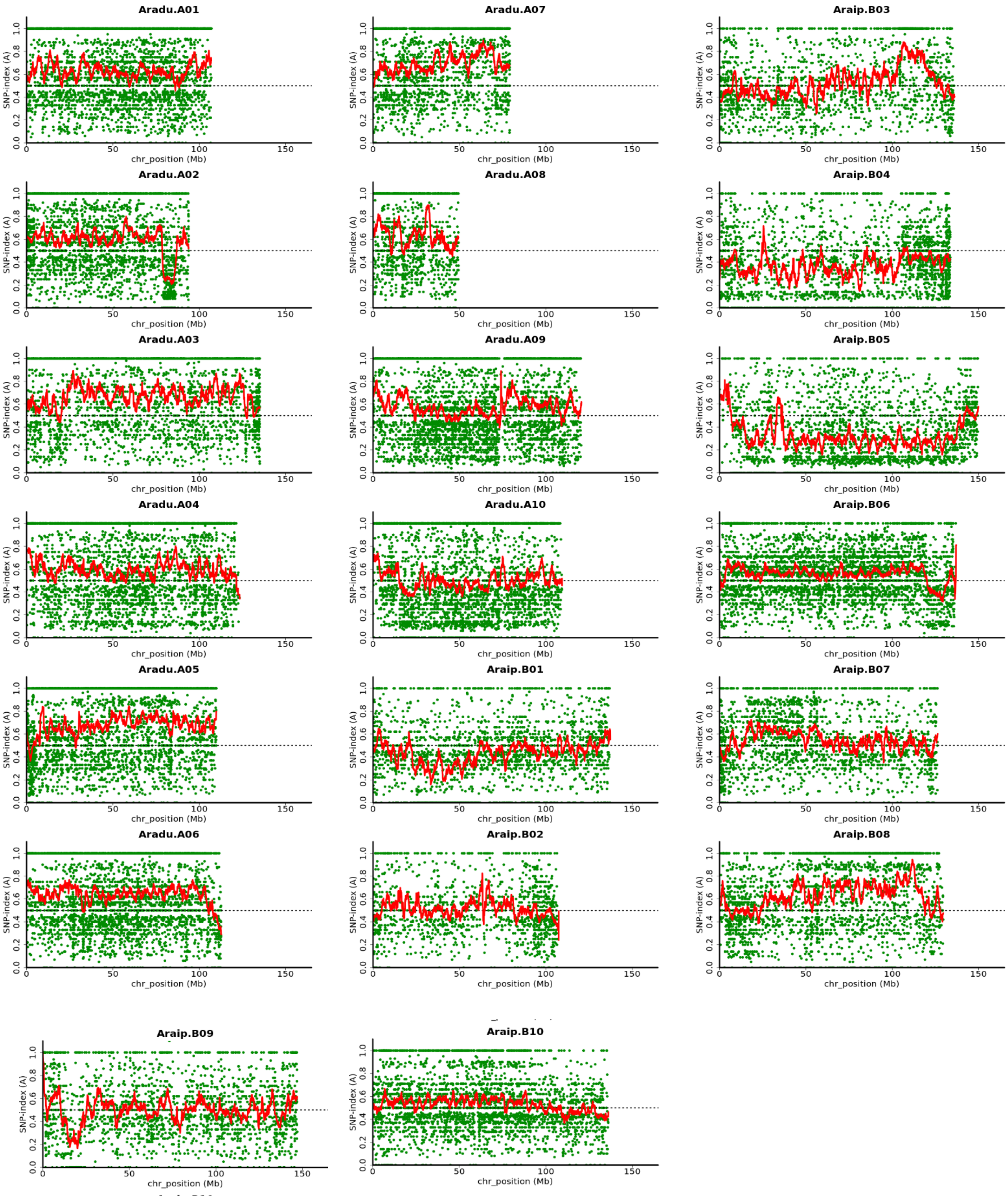

Supplementary Figure S3. SNP-index plots for 20 pseudomolecules Dormant pool with the dormant parent. Red lines indicate the sliding window average of 2 Mb interval with 50 kb increment for SNP-index.

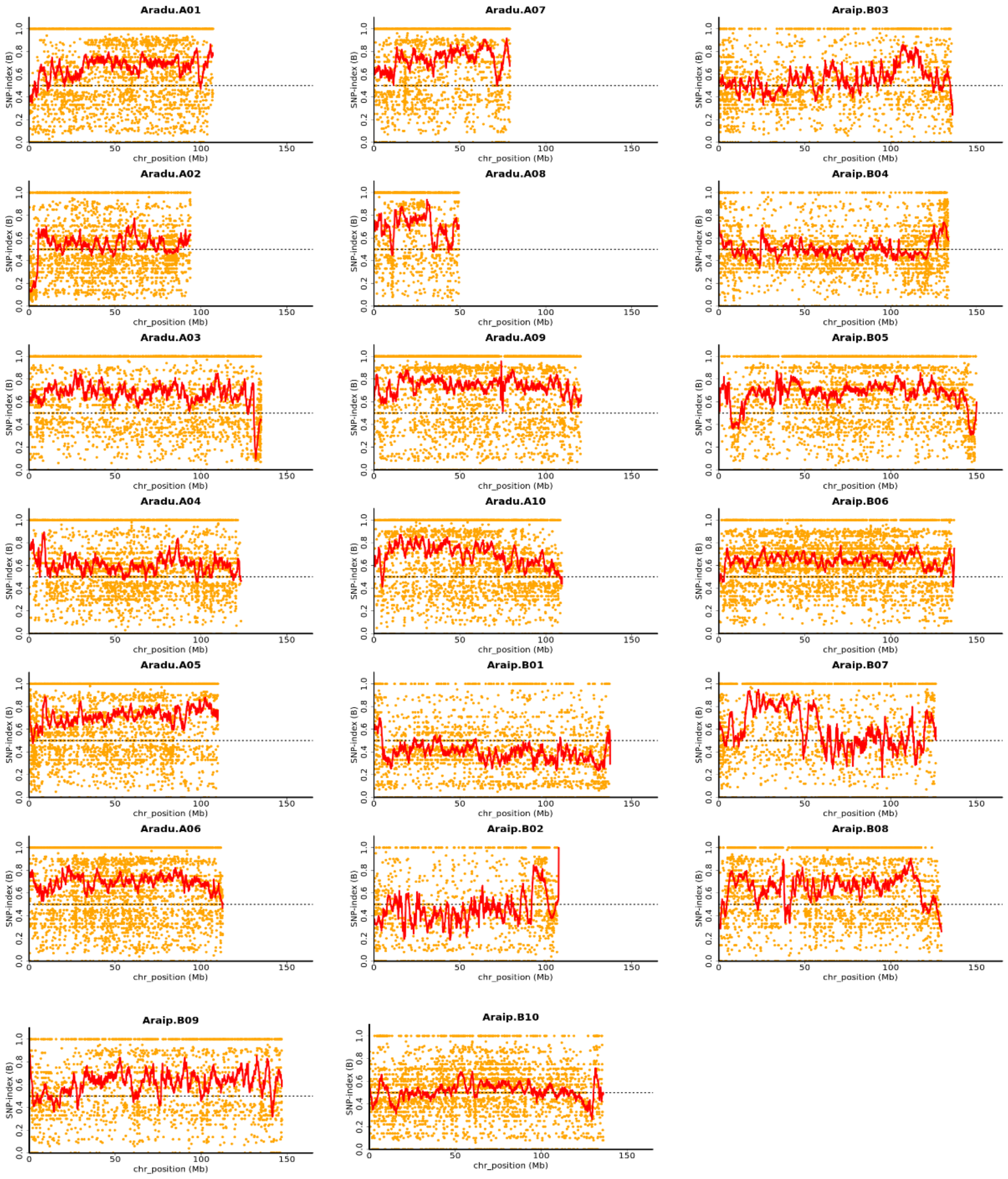

Supplementary Figure S4. SNP-index plots for 20 pseudomolecules of Non–dormant pool with dormant parent. Red lines indicate the sliding window average of 2Mb interval with 50 kb increment for SNP-index.

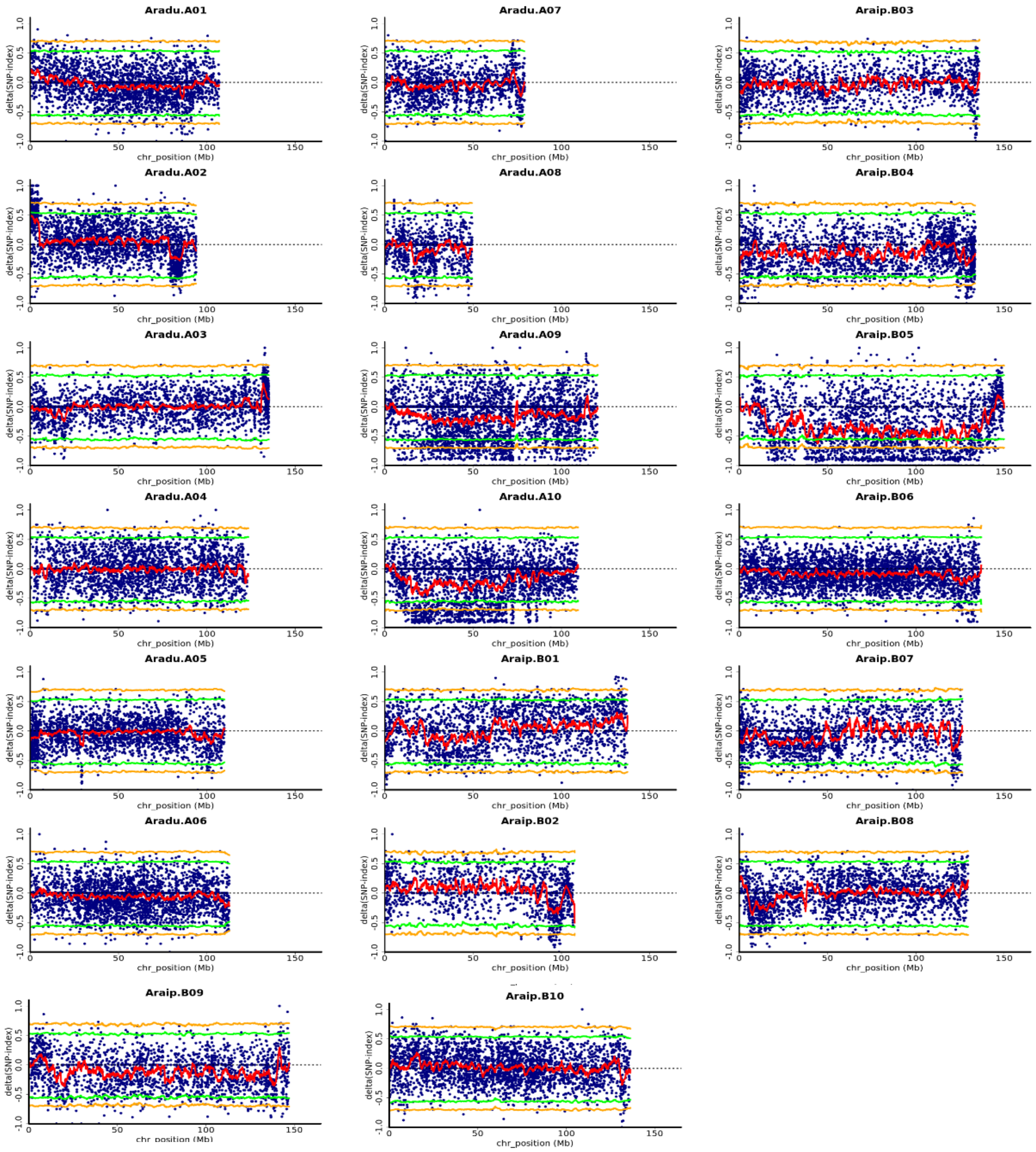

Supplementary Figure S5. The Δ(SNP index) plot obtained by subtraction of dormant pool SNP-index from non-dormant pool SNP-index Statistical confidence intervals under the null hypothesis of no QTL are shown (gray: $P < 0.1$; green: $P < 0.05$; pink: $P < 0.01$).
